# Could Metabolism-Related Long Non-Coding RNAs Be More Conserved than Their Brain-Related Counterparts?

**DOI:** 10.3390/genes17040484

**Published:** 2026-04-18

**Authors:** Laurent Metzinger, Valérie Metzinger-Le Meuth

**Affiliations:** 1 HEMATIM UR 4666, C.U.R.S, University of Picardie Jules Verne, CEDEX 1, 80025 Amiens, France; valerie.metzinger@univ-paris13.fr; 2 INSERM UMRS 1148, Laboratory for Vascular Translational Science (LVTS), UFR SMBH, University Sorbonne Paris Nord, 93000 Bobigny, France

**Keywords:** long non-coding RNA, metabolism, phylogeny, brain

## Abstract

The human genome produces a large repertoire of non-coding RNAs (ncRNAs) with important regulatory roles in development, physiology, and most of diseases. Among these, long non-coding RNAs (lncRNAs) have emerged as key modulators of gene expression, chromatin organization, and cellular homeostasis, despite displaying remarkably low primary-sequence conservation across species. This apparent evolutionary paradox questions the limitations of predicting biological function based on conservation, particularly across different biological domains. Here, we examine current evidence on lncRNA evolution, with a focus on their roles in metabolic regulation compared with neurobiological processes. We hypothesize that lncRNAs involved in ancient and conserved pathways such as metabolism may be under stronger evolutionary constraint than those associated with higher-order, species-specific traits, although available data support a more nuanced interpretation. Functional importance often correlates poorly with linear sequence conservation and instead appears to depend on higher-level features, including RNA secondary or tertiary structure, genomic context, regulatory architecture, and interactions with conserved molecular partners. We propose a systematic comparative framework to empirically assess conservation among metabolism- and neuro-associated lncRNAs using phylogenetic, syntenic, structural, and expression-based metrics. Finally, we discuss the therapeutic implications of lncRNA biology, highlighting how a deeper understanding of their evolutionary and mechanistic properties may inform the development of more precise and effective RNA-targeting strategies. Together, these insights underscore the non-coding transcriptome as a critical frontier for both fundamental biology and precision medicine.

## 1. Introduction

The relevance of the non-coding genome was a big surprise. For decades, vast regions of the human genome that do not encode proteins (constituting nearly 98% of total genomic content) were dismissed as “junk DNA” or genomic “dark matter”. However, this perspective has been profoundly revised. It is now clear that much of this non-coding space is actively and dynamically transcribed into a wide variety of ncRNA species, many exhibiting highly cell-type-specific and developmentally regulated expression patterns [1,2]. Non-coding RNAs (ncRNAs) have emerged as a major frontier in molecular biology and biomedical research. The biological and biomedical significance of these transcripts is increasingly evident, particularly as high-throughput transcriptomics and functional genomics begin to uncover their roles in development, cellular homeostasis, and diseases [3].

Among ncRNAs, microRNAs (miRNAs) and long non-coding RNAs (lncRNAs) have been the most extensively studied. A growing number of disease-associated genomic loci, including those identified through genome-wide association studies (GWAS), map to non-coding regions—providing compelling evidence that ncRNAs contribute directly to human pathologies [4]. Moreover, ncRNAs appear to act as key regulators of chromatin states, epigenetic trajectories, and transcriptional networks, frequently in cooperation with proteins harboring intrinsically disordered regions, which dominate known gene-regulatory protein classes [4,5,6,7].

**Figure 1:**
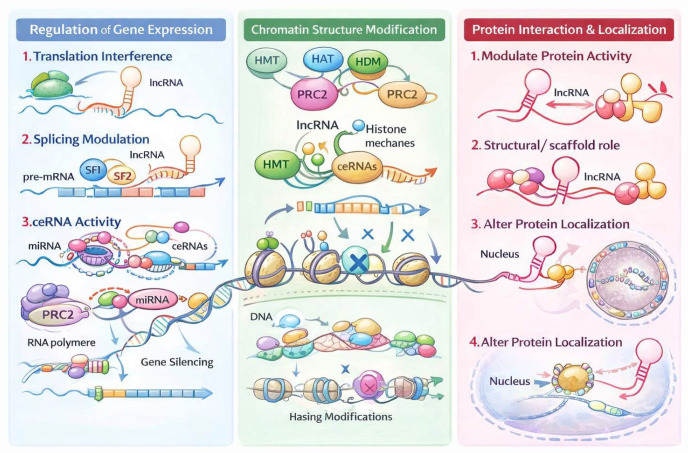
lncRNA biogenesis and various mechanisms of action. This illustration shows how lncRNAs are transcribed from non-coding genomic regions, processed (capping, splicing, polyadenylation or non–polyadenylation), and then engage in regulatory mechanisms such as chromatin remodeling, RNA–protein interactions, or transcriptional modulation. Abbreviations: ceRNA, competing endogenous RNA; HAT, histone acetyltransferase; HDM, histone demethylase; HMT, histone methyltransferase; lncRNA, long non-coding RNA; miRNA, microRNA; mRNA, messenger RNA; pre-mRNA, precursor messenger RNA; PRC2, Polycomb Repressive Complex 2; SF1, splicing factor 1; SF2, splicing factor 2.

Given their abundance (with lncRNAs estimated at 191,079, greatly outnumbering the 7563 short RNAs, GENCODE version 49, https://www.gencodegenes.org/human/stats.html, (accessed on 1 January 2026)) and their pronounced tissue specificity, lncRNAs represent a particularly promising reservoir of biomarkers and therapeutic targets (Figure 1). There are difficulties in determining which lncRNAs are functional, as estimates vary substantially depending on whether they are derived from computational predictions or experimental evidence. For example, work from the FANTOM Consortium used multiple metrics to assess functionality and suggested that approximately 60–70% of lncRNAs may be potentially functional [3]. In contrast, subsequent experimental-based estimates from the same consortium placed this number in a considerably lower range, around 20–30% in human fibroblasts [8]. This apparent discrepancy may, however, simply reflect the limited scope of current experimental evidence, as a given lncRNA’s function depends not only on cell type but also on cell state, disease context, cellular activation, and environmental stimuli. Addressing these complexities will likely require more thorough and systematic computational approaches, as proposed here, in combination with broader experimental frameworks. Several ncRNAs have already been linked with clinical outcomes—for instance, in chronic kidney disease [9,10]—offering preliminary proof-of-concept for their translational value. The present challenge is to develop efficient and highly specific strategies for manipulating ncRNA function in vivo.

One of the most striking and somewhat paradoxical features of lncRNAs is their relatively poor primary-sequence conservation across species, despite mounting evidence for functional relevance. Unlike protein-coding genes—restrained by codon structure and amino-acid requirements—lncRNAs often evolve rapidly, accumulating substitutions with little apparent detriment to their functional roles [11]. This phenomenon reflects the fact that many lncRNAs exert their biological activity through mechanisms that do not rely on strict primary-sequence conservation. Instead, their functions often depend on higher-order RNA secondary or tertiary structures; short, modular sequence motifs, or RNA–protein/RNA–DNA/RNA–chromatin interactions, all of which can be maintained even when the underlying nucleotide sequence diverges substantially [12]. As a result, many lncRNAs have only limited stretches of detectable similarity across distantly related species, and in some cases, no clear orthologs can be identified at all. Some lncRNAs even appear to arise de novo from previously untranscribed genomic regions, giving rise to lineage-specific regulatory transcripts [13]. On the other hand, a subset of lncRNAs demonstrates deep evolutionary conservation, including notable examples such as XIST, H19, and MALAT1 [11]. Thus, examples such as MALAT1 and NEAT1, which are broadly studied and implicated in cancer and many other biological contexts, may not be representative of any specific lncRNA population. Moreover, a small number of well-studied examples is insufficient to draw conclusions that contradict broader trends in lncRNA evolution. These cases illustrate that, when structural or functional constraints are sufficiently stringent, selection can preserve both expression and architecture across long evolutionary distances. Still, these conserved transcripts represent a minority within the vast lncRNA repertoire.

In this work, we aimed to contrast two biological systems that differ in well-established evolutionary and functional properties. Core metabolic pathways are among the most ancient and broadly conserved components of cellular biology, shared across nearly all forms of life due to their essential role in energy production and basic homeostasis. As a result, metabolic genes and processes are often expected to show strong evolutionary constraint across species. In contrast, the brain was chosen as a paradigm of a tissue system characterized by high cellular diversity, complex regulatory architecture, and lineage-specific adaptations [14]. Neural and cognitive traits are known to evolve rapidly in certain clades, driven by ecological and behavioral pressures, and are frequently associated with expanded gene regulatory innovation rather than strict conservation of molecular components.

## 2. lncRNAs in Metabolic Regulation

Emerging evidence implicates lncRNAs as important regulators of glucose and lipid metabolism. Studies in mammalian models and human tissues have demonstrated that lncRNAs can modulate central metabolic pathways under both physiological and pathological circumstances [15]. Deregulation of specific lncRNAs has been associated with metabolic disorders such as insulin resistance [16], steatosis [17], or dyslipidemia [18]—suggesting their potential functional relevance. Interestingly, lncRNAs that display higher evolutionary conservation tend to possess features often associated with regulatory constraint: longer gene bodies, more exons, a greater number of transcript isoforms, broader expression across tissues, and a stronger association with disease phenotypes [3]. This observation raises the logical hypothesis that lncRNAs involved in fundamental metabolic processes—which are also ancient and highly conserved across eukaryotes [19]—might be under stronger selective pressure, and thereby more conserved than lncRNAs involved in higher-order, species-specific traits such as cognition. This hypothesis draws support from the conservation of core metabolic pathways and the regulatory proteins that govern them (e.g., metabolic enzymes, transcription factors, miRNAs) [20]. The expansion of non-coding sequences in vertebrate genomes, as highlighted by the so-called “C-value enigma,” [21] further suggests that regulatory innovation—particularly in complex tissues such as the brain—may be propagated through lineage- or species-specific lncRNAs.

## 3. lncRNAs in Cerebral Activity

We use the brain in this work as an illustrative comparator because, unlike core metabolic pathways that are deeply conserved across taxa, neural systems exhibit high regulatory complexity and have undergone substantial lineage-specific diversification associated with cognition and behavior. This contrast provides a useful framework for examining how evolutionary constraint differs between ancient housekeeping functions and more specialized, rapidly evolving traits. Indeed, the neural tissues require finely tuned gene expression programs to coordinate the development and function of numerous specialized cell types. Certain evolutionary lineages exhibit accelerated changes in neural and cognitive features, driven by environmental and behavioral factors, and this flexibility is often associated with novel regulatory developments instead of strong molecular conservation [14]. In this context, lncRNAs have emerged as key contributors to regulatory diversification, as they can modulate gene expression through interactions with chromatin, transcription factors, and RNA-binding proteins. Many lncRNAs display strong tissue- and cell-type specificity in the brain, supporting their role in shaping lineage-specific regulatory networks. Moreover, the rapid evolutionary turnover of lncRNAs suggests that they may provide a substrate for adaptive changes in neural gene regulation, ultimately contributing to the emergence of novel cognitive and behavioral traits. One could thus hypothesize that metabolism-related lncRNAs could be more conserved than their brain-related counterparts.

## 4. Limitations of Predicting Biological Function Based on Conservation

Despite the intuitive appeal of the preceding hypothesis, evidence from comparative genomics and evolutionary transcriptomics supports a more nuanced interpretation. While GWAS frequently implicate noncoding loci in disease susceptibility [22], evolutionary conservation patterns of lncRNAs have been primarily characterized through comparative genomics approach [23]. Key findings include that (i) the majority of lncRNAs exhibit minimal primary-sequence conservation, regardless of their putative physiological roles [3]. Conservation that matters may thus rest at higher organizational levels—such as promoter sequences, splice junctions, syntenic genomic context, or RNA structural domains—rather than on linear nucleotide identity. (ii) Several lncRNAs with essential neuronal or developmental functions (e.g., MALAT1, NEAT1, MEG3, BC200/BC1) display evidence of structural conservation, constrained promoter architecture, or preserved expression patterns across species—contradicting the assumption that neuro-associated lncRNAs must generally be lineage-specific [24,25], And (iii) lncRNA evolution appears to favor flexibility and adaptability, with many transcripts evolving rapidly, perhaps in response to lineage-specific selective pressures or novel physiological demands [3,23]. This suggests that functional relevance does not always correlate with strong sequence conservation.

Taken together, these observations suggest that evolutionary constraint in lncRNAs may depend less on the broad biological domain in which a transcript operates (e.g., metabolism versus neurology) and more on specific molecular features such as mechanism of action (scaffold, decoy, guide), requirements for conserved RNA structure, genomic context, and interactions with conserved protein partners [26]. However, we note that this remains an emerging hypothesis rather than a settled rule, and systematic comparative analyses will be required to directly evaluate which of these features best predict conservation across functional categories.

## 5. Proposed Strategy for Empirical Evaluation

**Figure 2:**
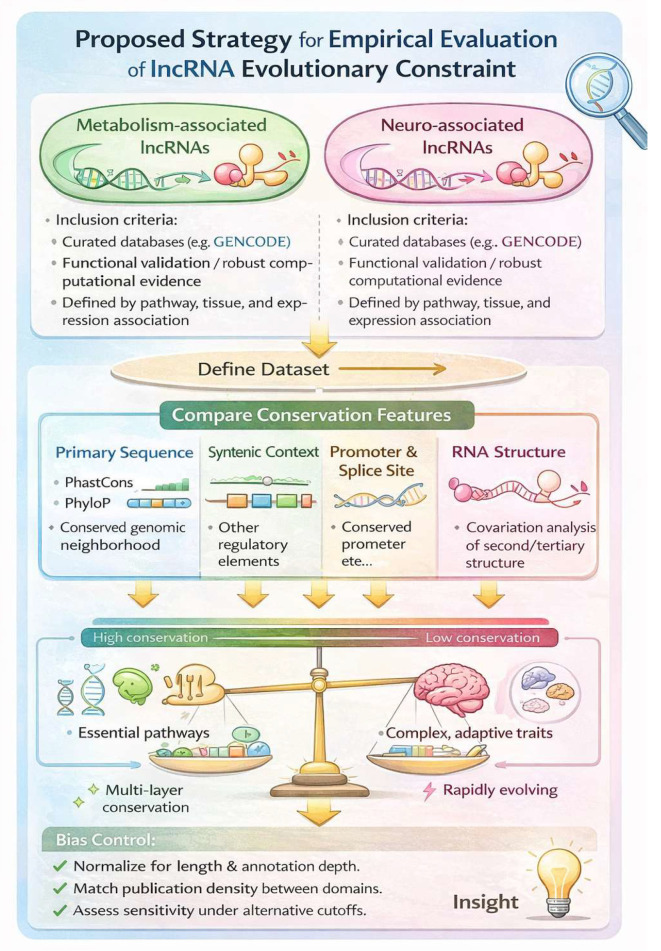
*Proposed Strategy for Empirical Evaluation*. Metabolism- and neurobiology-associated lncRNAs will be selected using curated annotation resources (e.g., GENCODE) and functional evidence from the literature and pathway databases. Inclusion criteria require either experimentally validated functional annotation or strong computational prediction supported by multiple independent studies. LncRNAs with ambiguous or conflicting functional assignments will be excluded. Functional attribution will be based on pathway enrichment, GO terms, or tissue-specific expression patterns, ensuring consistent and evidence-based group assignment.

A major unresolved question in evolutionary transcriptomics is whether lncRNAs involved in deeply conserved physiological systems (such as metabolism) experience stronger evolutionary constraint than those associated with more lineage-adaptive systems (such as neurobiology). Addressing this requires an empirical framework that goes beyond primary-sequence identity and instead integrates multiple layers of conservation, including genomic context, regulatory architecture, structural constraints, and expression preservation (Illustrated in Figure 2).

### 5.1. Defining Functional lncRNA Sets Across Biological Domains

The first step is the assembly of high-confidence lncRNA sets associated with metabolism and neurobiology. Rather than relying solely on tissue-enriched expression, inclusion should be based on functional evidence, such as experimentally validated roles in metabolic regulation (e.g., lncRNAs implicated in insulin signaling, lipid homeostasis, or mitochondrial pathways) or neurodevelopmental processes (e.g., synaptic regulation, neuronal differentiation). Curated annotation resources such as **GENCODE**, **LNCipedia**, and **NONCODE** provide transcript models, while pathway association can be derived from **Gene Ontology**, **KEGG**, and Reactome-based enrichment. Importantly, functional assignments must be supported either by mechanistic studies or multiple independent datasets, reducing the risk of annotation noise.

### 5.2. Multi-Layered Conservation Metrics Beyond Nucleotide Identity

A central point of the “lncRNA conservation paradox” is that functional constraint may not be reflected in linear sequence similarity. Therefore, comparative analyses should incorporate several orthogonal conservation layers:

- **Primary sequence constraint** can be quantified using PhastCons and PhyloP scores derived from multi-species alignments [27], as well as GERP++ scores to detect rejected substitutions [28]. These metrics allow detection of conserved elements within exons or short functional motifs.
- **Promoter and enhancer conservation may** better capture selective pressure acting on transcriptional regulation rather than RNA sequence itself. They may be assessed using cross-species alignment of regulatory regions combined with chromatin-state conservation derived from comparative ATAC-seq or ChIP-seq datasets. Tools such as LiftOver enable mapping of promoter coordinates across genomes [29], while conservation of transcription factor binding sites can be evaluated using position-weight matrix (PWM) analysis across aligned promoter regions.
- **Splice-site and exon–intron architecture conservation** can indicate preserved transcript processing, even when exon sequences diverge. They can be evaluated by comparing exon boundaries across species using genome alignments (e.g., via Ensembl Compara). Tools such as CESAR (Coding Exon-Structure Aware Realigner) and splicing-aware aligners allow assessment of conserved splice donor/acceptor sites [30]. Conservation of intron phase, exon number, and transcript isoform structure provides evidence of selective constraint even when exon sequences diverge.
- **Syntenic conservation,** defined by preserved genomic neighborhood, is particularly important for rapidly evolving lncRNAs lacking clear orthologs. It can be analyzed using pairwise or multi-species synteny blocks derived from Ensembl Compara or MCScanX. For rapidly evolving lncRNAs lacking clear sequence orthology, conserved flanking protein-coding genes can provide evidence of positional orthology [31,32].

Such approaches allow classification of lncRNAs into distinct evolutionary modes: sequence-conserved, regulatory-conserved, structure-conserved, or lineage-specific.

### 5.3. Structural Constraint as a Major Driver of lncRNA Conservation

Many lncRNAs act through RNA secondary or tertiary structures rather than through encoded peptides or a strict motif. Thus, conservation should also be evaluated at the structural level using covariation-based methods and comparative folding approaches. For example, deeply conserved lncRNAs such as **MALAT1** and **NEAT1** retain conserved structural domains despite modest overall sequence conservation, suggesting selection on RNA architecture. Integrating structure-aware conservation metrics may therefore reveal hidden constraints missed by standard alignments. Because many lncRNAs act through RNA secondary or tertiary structures rather than primary sequence motifs, conservation should be evaluated at the structural level using comparative and covariation-based approaches. Tools such as RNAz and EvoFold detect thermodynamically stable and evolutionarily conserved RNA secondary structures across multiple alignments [33]. Covariation analysis using R-scape can identify compensatory base changes indicative of selection acting on RNA structure [1,34]. Infernal and covariance models (CMs) further enable structure-aware homology searches that detect conserved structural domains even when linear sequence similarity is weak.

Comparative folding strategies, integrating minimum free energy predictions with cross-species alignments (e.g., via MAFFT or Clustal Omega), can reveal structurally constrained domains embedded within otherwise rapidly evolving transcripts [35,36]. Such structure-informed metrics may uncover hidden evolutionary constraints that is not detectable through conventional nucleotide identity thresholds.

### 5.4. Expression Conservation and Functional Analogy Across Species

Because many lncRNAs are tissue-specific, expression conservation provides an additional axis of evolutionary constraint. Comparative transcriptomic atlases across vertebrates can be used to assess whether orthologous loci exhibit conserved developmental timing, tissue restriction, or stimulus responsiveness. Notably, conservation of expression patterns may persist even in cases where direct orthology is unclear, supporting the possibility of functional analogs rather than strict sequence orthologs. Cross-species RNA-seq datasets can be normalized using TPM-based approaches or variance-stabilizing transformations to enable interspecies comparisons. Conservation of expression can then be quantified using correlation-based metrics across matched tissues, developmental stages, or physiological conditions. Tissue specificity can be assessed using indices such as the Tau specificity index, enabling comparison of the breadth of expression across species [37]. In addition, emerging cross-species single-cell transcriptomic integration frameworks (e.g., ortholog-guided clustering or mutual nearest neighbor approaches) allow evaluation of whether lncRNA loci exhibit conserved cell-type–restricted expression patterns.

Importantly, expression conservation may persist even when strict sequence orthology is unclear, raising the possibility of functional analogs rather than direct orthologs. Integrating positional conservation, structural features, and cross-species expression similarity therefore provides a more comprehensive view of lncRNA evolutionary constraint.

### 5.5. Controlling for Annotation Bias and Domain-Specific Overrepresentation

A major challenge is that neuro-associated lncRNAs are disproportionately studied, leading to annotation depth bias. Metabolism-related lncRNAs may appear less conserved simply because fewer have been characterized. To mitigate this, metabolism- and neuro-lncRNA groups should be matched for transcript length, exon count, expression breadth, and publication density. Sensitivity analyses under alternative inclusion thresholds can further test robustness.

### 5.6. Expected Outcomes and Interpretation

If metabolism-associated lncRNAs exhibit higher conservation across multiple layers (promoter, synteny, structure, expression), this would support the hypothesis that essential homeostatic pathways impose stronger evolutionary constraint on regulatory noncoding transcripts. Conversely, if both groups show similarly weak sequence constraint but differ in regulatory or structural preservation, this would suggest that lncRNA evolution is shaped less by pathway category and more by molecular mechanism of action (guide, scaffold, decoy) and interaction with conserved protein complexes. Overall, this framework provides a testable strategy to resolve whether evolutionary conservation in lncRNAs reflects biological domain, mechanistic requirement, or regulatory architecture. This approach could help clarify whether and to what extent evolutionary constraint correlates with functional relevance in different biological domains.

## 6. Therapeutic Implications

Taken together, these considerations highlight that evolutionary conservation in lncRNAs cannot be inferred solely from broad functional categories such as metabolism or neurobiology, but instead reflects a complex interplay of structural constraints, genomic context, and molecular mechanism. Importantly, this issue is not only of theoretical interest for evolutionary transcriptomics, but also carries direct translational relevance. Indeed, understanding which lncRNAs are conserved, which are lineage-specific, and which molecular features are maintained across species has major implications for functional prioritization, experimental modeling, and ultimately therapeutic targeting. As efforts to manipulate the non-coding transcriptome advance, integrating evolutionary and mechanistic insight will be essential for identifying robust disease-associated lncRNAs and for designing interventions that achieve both specificity and efficacy in vivo. So, a better understanding of lncRNA functions and phylogeny will help to develop efficient and highly specific strategies for manipulating ncRNA function in vivo. Although the therapeutic potential of ncRNAs has only recently gained broad recognition, several clinical trials (Phase I/II) targeting miRNAs—e.g., in diabetes, fibrosis, hepatitis—are already ongoing. Current ncRNA-targeting strategies rely largely on antisense-based modalities [38] (antisense, siRNAs, mimics), which, while effective in theory, remain limited by off-target effects and suboptimal specificity [39]. The advent of RNA-targeting CRISPR systems (e.g., Cas13) [40] offers a promising alternative, with the potential for more precise, programmable, and tunable intervention. Expanding these approaches to include lncRNAs could unlock a vast, largely untapped repertoire of therapeutic targets, particularly given their tissue-specific expression patterns and involvement in diverse pathological processes. Considering the enormous number and regulatory versatility of lncRNAs, the non-coding transcriptome may well represent the “next frontier” in precision medicine, especially for complex, chronic, or multifactorial diseases.

## 7. Conclusions/Limitations of the Study

In this work, we ask whether long non-coding RNAs (lncRNAs) associated with fundamental metabolic processes could exhibit greater evolutionary conservation than those involved in neurobiological functions. To address this, we propose a framework offering a testable hypothesis linking evolutionary constraint to the fundamental nature of the underlying biological processes. However, several limitations must be acknowledged. First, the approach remains highly dependent on the quality and completeness of current annotations, which are uneven across tissues and biological domains and may introduce systematic bias—particularly given the relative overrepresentation of neuro-related lncRNAs in the literature. Second, deducing functional conservation from indirect metrics such as synteny, structure, or expression similarity does not necessarily establish mechanistic equivalence and may overlook lineage-specific functional divergence. Third, current computational tools for structural prediction and cross-species transcriptomic integration remain imperfect, especially for long and lowly expressed transcripts, potentially limiting sensitivity and reproducibility. Finally, the classification of lncRNAs into functional categories (such as metabolic versus neurobiological) may itself be reductive, as many lncRNAs participate in pleiotropic regulatory networks spanning multiple physiological systems. Together, these considerations emphasize that while the proposed framework provides a useful and testable strategy to investigate lncRNA evolution, its conclusions will require careful interpretation and experimental validation.

## Data Availability

No new data were created or analyzed in this study. Data sharing is not applicable to this article.
